# Mixed-Method, Multilevel Clustered-Randomized Control Trial for Menstrual Health Disparities

**DOI:** 10.1007/s11121-024-01646-1

**Published:** 2024-02-15

**Authors:** Lauren C. Houghton, Paris B. Adkins-Jackson

**Affiliations:** 1 Department of Epidemiology, Mailman School of Public Health, Columbia University, New York, USA; 2 Department of Sociomedical Sciences, Mailman School of Public Health, Columbia University, New York, USA

**Keywords:** Menstrual health, Critical race theory, Intersectionality, Structural racism, Mixed methods, Cluster randomized control trial

## Abstract

Menstrual cycle characteristics are largely considered unmodifiable reproductive factors, a framing that prevents exploration of the ways structural factors interfere with menstrual health. Given the role of structural factors like healthy food and healthcare access on reproductive health and the grave need for structural interventions to known reproductive health disparities that disproportionately target cisgender women racialized as Black, it is imperative that science begin to examine how structural factors influence menstrual health. To explore such research, we employ critical race theory and intersectionality to illustrate what a structural intervention to improve menstrual cycle health could look like. Centering those with the greatest need, persons racialized as Black and/or LatinX living in food and healthcare deserts in Northern Manhattan, our illustrative sample includes four groups of persons who menstruate (e.g., cisgender girls and women) that are pre-menarche, pre-parous, postpartum, or perimenopausal. We describe a hypothetical, multilevel clustered-randomized control trial (cRCT) that provides psychoeducation on racism-related trauma and free delivered groceries to both treatment and control groups, while randomizing 30 clusters of housing associations to receive either sexual health clinics at their housing association or free vouchers for healthcare. We embed mixed methods (diaries, interviews, surveys, mobile apps, observation) into the design to evaluate the effectiveness of the 1-year intervention, in addition to determining the impact on participants through their perspectives. Through this illustration, we provide a novel example of how structural interventions can apply mixed methods to evaluate effectiveness while delivering services to populations impacted by multiple structural factors. We demonstrate how qualitative and quantitative approaches can be paired in clustered RCTs and how a living logic model can empirically incorporate the population perspective into more effective interventions. Lastly, we reveal how sensitive menstrual health is to structural factors and how upstream improvements will trickle down to potentially reduce health disparities in reproductive health.

## Introduction

There is a robust body of literature that demonstrates how structural factors, like lack of access to healthy food and healthcare, determine health. Groups assigned a privileged value in a stratified society are given disproportionate access to foods and healthcare that can sustain good quality health and improve health in times of precarity. This differential treatment and access produce health disparities, which in the context of the United States (US), is blatantly observable in racial and gender health disparities in disease incidence, morbidity, and mortality. The intersectional experience of both racism and sexism is emblematic in the case of menstrual health, a vital sign of health (“Menstruation in Girls and Adolescents: Using the Menstrual Cycle as a Vital Sign,” 2021) for persons who menstruate (PWM) like cisgender girls and women.

Menstrual cycle characteristics consist of the age when PWM first menstruate (menarche), last menstruate (menopause), and cycle regularity in between (cycle length over time). There are stark racial disparities that exist for reproductive health outcomes (Kjerulff et al., 1993). For example, the most recent national surveys suggest that PWM racialized as LatinX have younger menarche than PWM racialized as non-LatinX White (*National Health Statistics Reports, Number 146, September 10, 2020*, 2020; Srikanth et al., 2023) and PWM racialized as Black and LatinX have earlier ages at menopause than PWM racialized as White (Appiah et al., 2021). In an observational sample using cycle tracking and survey data from the Apple Women’s Health Study, PWM racialized as LatinX have longer and more varied cycle lengths than PWM racialized as White (Boston & Ma 02115, 2022). The differences in age at menarche and menopause and cycle regularity are important because these characteristics are associated with chronic diseases like endometriosis, polycystic ovary syndrome, breast cancer, and cardiovascular disease, thus, exacerbating existing health disparities (Blanken et al., 2022; Golub et al., 2008; Titus-Ernstoff et al., 1998). As critical race public health praxis and intersectional frameworks suggest (Ford & Airhihenbuwa, 2010a), racial and gender disparities are only downstream effects to differential treatment and access resulting from structural racism and sexism. These same downstream effects can be observed in disparities in healthcare literacy and utilization. Structural racism and sexism directly interfere with individual-level agency and resilience that could be employed to potentially lessen the blow of other structural determinants. Despite individual-level modifications to menstrual health, individual-level agency and resilience through healthcare literacy and utilization may not overcome the impact of structural determinants like lack of access to healthy food and healthcare. The female reproductive system is highly sensitive to environmental factors, and sudden changes in these factors can impact menstrual cycle characteristics month to month, and further, prolonged environmental stressors affect menstrual health throughout the life course (Ellison et al., 1993). Subsequently, it is more impactful over the life course to change upstream structural influences like food and healthcare access, in addition to the myriad of downstream implications like healthcare literacy and utilization.

This article is an illustration of a hypothetical structural intervention that addresses multiple levels: individual behaviors, community resources, and healthcare systems. In the following illustration, we propose that the menstrual health of PWM racialized as Black, LatinX, and other minoritized groups living in Northern Manhattan can be improved by testing a multilevel intervention via clustered-randomized control trial (cRCT) and evaluating it through mixed methods. The intervention has the potential to disrupt the impact of lack of trauma-informed and sexual health literacy, as well as healthy food and healthcare access on menstrual health for PWM across the life course, especially for those that experience the greatest burden of reproductive health disparities.

### Clustered RCTs

Though there is a dearth in the literature describing interventions to structural racism, there are meaningful shifts occurring that reimagine interventional approaches like RCTs. Traditionally, RCTs focus on individual-level factors and do not address structural factors. Additionally, RCTs rely on quantitative outcomes to assess effectiveness. Despite these norms in clinical trials, there are modifications that can be made to improve the utility of approaches like the multilevel clustered RCT. Clustered RCTs, also known as group randomized trials, randomize entire clusters of people rather than individual participants (Murray et al., 2004). Multilevel clustered RCTs require a mixture of intervention levels. While the unit of randomization may be at the community level, the intervention may be delivered at the institution, community, and/or individual levels. This multilevel approach presents an opportunity to strengthen the validity of intervention science.

When conducting clustered RCTs in historically marginalized communities, there is the risk that researchers inadvertently contribute to disparities under the guise of robust study design. For example, a researcher may choose to offer the intervention to one arm of the study and fervently defend this practice of science as an approach to test whether the intervention improved the outcomes of persons who received it apart from those that did not. Such an approach is extremely problematic when the intervention is potentially lifesaving or otherwise a human necessity. This is a recurring conundrum for clinical trial research with high-impact treatments. Despite this cyclical challenge, there are more ethical and equitable ways to implement RCTs.

An alternative is to deliver resources through intervention components across multiple levels to both intervention and control arms and manipulate the delivery of one intervention component at only one of the levels. For example, in our illustrative study, we provide both arms healthcare access: the intervention arm will receive sexual healthcare at their public housing association with a community health worker to assist with navigation; the control arm will receive community health workers that will assist with dispensing vouchers and setting up sexual healthcare appointments for participants. Through this illustration, everyone receives resources because in the case of multiple structural determinants influencing health, it is not equitable to provide resources of the intervention to only some and not others. Doing so reinforces inaccessibility and positions science as complicit in the inequitable distribution of resources that plagues many stratified societies.

### Mixed-Method Approaches

In addition to providing resources to both arms, cRCTs must embrace mixed methods. Multi-method refers to the use of multiple data collection strategies in research. Mixed methods is a multi-method approach that combines diverse data generally categorized as qualitative and quantitative (Creswell & Plano Clark, 2018). Qualitative data are words and phrases obtained from data collection strategies like interviews and open-ended responses on surveys. These data require appropriate analytic strategies, some of which include constant comparison and content analysis. Quantitative data are responses that can be linked to a corresponding numeric value. Quantitative data can be obtained from data collection strategies like observation, biomarkers, surveys, and others. These data also demand specialized analytic strategies, of which the most used are statistics. When qualitative and quantitative data are integrated in a study, these mixed methods expand the range of participant perspectives available. For a multilevel cRCT, a mixed-method approach introduces qualitative data through the detailed perspectives of participants on the experience and impact of the intervention.

Along with diverse data, the use of qualitative data invites interventionists and other researchers to engage methodological frameworks that provoke practices of reflexivity, the process of making oneself aware of the unconscious biases held and the ways those biases may influence decision-making and interpretation of the research (Finlay, 2002). Like a theoretical framework shapes the relationship between the exposure and the outcome, a methodological framework shapes the selection and application of methods and analyses, which requires reflexivity. For example, the study team may discuss the reasons why they are drawn to the study topic, proposed design, and study methods to reflect in concert with each other on where biases exist and how the study team can be responsive to it (Hardeman & Karbeah, 2020). The reflexive process also includes an examination of the underlying ontological assumptions and history of the methods and analytic strategies proposed.

For example, quantitative data, validated through statistical approaches, can carry a post-positivist assumption that the data obtained and analyzed represents a true value of an experience. Contrarily, qualitative data, via a social constructivist perspective, assumes there is not a true reality, only a co-created value contextualized by participants and their interaction with the study and team. Much like the concept of error in statistics, many qualitative approaches presume there are differences between the true value and what is captured. The exception being that qualitative methods generally reside at this difference, while quantitative methods continuously seek to bridge the gap in order to apply sample-specific data to the population level. Historically, the application of sample-specific characteristics to populations has had deleterious effects for populations minoritized in society (e.g., race-based algorithms for health). By scrutinizing the intentions, biases, history, trainings, and assumptions of a study team and a study’s proposed methods, interventionists can design mixed-methods research in ethical and respective ways. The commitment to engaging reflexive processes in mixed-methods research strengthens the design and implementation of a multilevel cRCT while providing robust data on the whole experience of participants.

## Methods

In this illustration, we describe a hypothetical mixed-method, multilevel cRCT to intervene upon structural determinants of menstrual health for PWM living in Northern Manhattan, New York. We hypothesize that menstrual health via regular cycle length and later ages at menarche and menopause can be improved by increasing access to healthy food and trauma-informed education (literacy) on menstrual health and the effects of structural racism on health. Due to the interaction between structural determinants of health, we further hypothesize that by providing access to healthcare at the homes of participants, we will increase their access to sexual health services. This approach aligns with the convergence strategy for multilevel interventions (Weiner et al., 2012). While we are unable to tease apart the additive vs synergistic effects of each intervention component, we are measuring outcomes at different levels through quantitative and qualitative methods, which can contribute to understanding how the components of a multilevel intervention affect individual outcomes.

### Positionality

This article is authored by two interdisciplinary researchers, with prior experiences with mixed-method approaches, in response to a call for papers on “Design and Analytic Methods to Evaluate Multilevel Interventions to Reduce Health Disparities.” We were drawn to the call because we know from professional experience how embedding qualitative methods into quantitative studies enhances rigor and we wanted to develop a process to capture that enhancement. To be responsive to the call, we developed a hypothetical intervention that brings together aspects of our individual research interests to illustrate the utility of mixed methods to reduce health disparities in community settings. We both were trained in anthropology before epidemiology and statistics, which informs our understanding of the utility of mixed methods. Dr. Houghton is a PWM with firsthand experience of menstrual shame during adolescence and medical disregard of menstrual health issues during early adulthood. She is cisgender and multi-racial but presents as White and has not experienced menstruation as a marginalized person. This positionality, in addition to 15 years of studying menstrual health (Houghton & Elhadad, 2020), influences Dr. Houghton’s choices in designing this intervention as well as blind spots in its development in terms of race and socioeconomic position. Dr. Adkins-Jackson is a PWM who has firsthand experience with the impact of structural determinants on her menstrual cycle regularity. This includes how socioeconomic factors have forced her to be born and continuously reside in food and healthcare deserts, exacerbated by poor health literacy on menstrual health and racism-related stress, culminating in fluctuating menstrual health. These experiences, in addition to multidisciplinary training and location in the academia, influence study design. We are both cisgender women that have limited understanding of the experiences of PWM that do not identify as cisgender female. Therefore, this is a blind spot ripe with opportunity for bias. From these trainings, lived experiences, and biases, we have collaborated to envision the following illustrative paradigm. If this were a real-world example, we would begin the process by collaborating with community members to design the study. With our goal being an illustration, at this time, it is not ethical to ask community members to donate their time and effort for an academic exercise.

### Methodological Frameworks

*Critical race theory* is a theoretical and methodological framework for the examination of racial equity (Ford & Airhihenbuwa, 2010a). The theory descends from the scholarship of racialized and minoritized legal scholars like Derrick Bell, Kimberle Crenshaw, Richard Delgago, Mari Matsuda, and Patricia Williams, among others. Critical race theory was adapted for public health use by Drs. Chandra Ford and Collins Airhihenbuwa (Ford & Airhihenbuwa, 2010b). Like critical race theory, a public health critical race praxis encompasses a race consciousness to examine the manifestation of racism in topic(s) under study, the mechanisms by which racism may influence the topic(s), a centering of persons most impacted by racism’s role on the topic(s) that are likely on the margins of the discourse, and a praxis (e.g., reflexivity) that encourages an iterative critical examination of the above along with corresponding actions to implement change.

For this illustration, we adopt the practice of reflexivity, in addition to applying a critical examination of the role of structural racism in the allocation of structural determinants of health. It is structural racism that brings together determinants like literacy, food access, and healthcare access to amplify impact upon racialized and minoritized groups. Therefore, we have designed an illustration that tackles multiple determinants. It is structural racism that consolidates large communities of persons racialized as Black and/or LatinX in Northern Manhattan, and further, in particular housing associations. Federal, state, and local agencies administer public housing to provide subsidized assistance for low-income households. These complexes are operated by state and local housing authorities that are authorized and funded by the US Department of Housing and Urban Development. One of the many services housing associations provide is to connect residents to opportunities in financial empowerment, business development, career advancement, and educational programs. Therefore, we will offer the intervention at these housing association buildings. Additionally, we are intervening on institutional racism by providing structurally and culturally responsive training to healthcare. As critical race theory necessitates a centering of the margins, the margins have repeatedly informed the masses that there is a dire need for structural change over individual change. Engaging a public health critical race praxis helped us to develop a multilevel structural intervention that works within and outside of multiple institutions to modify structural determinants instead of individual behavior.

*Intersectionality*, also a theoretical and methodological framework, is intimately related to the origins of critical race theory as it shares a key scholar in its development, Dr. Kimberle Crenshaw (Carbado et al., 2013). More so, intersectionality descends from political critique from scholars and activists like Sojourner Truth, and later, the Combahee River Collective. Intersectionality in health has been shaped by Drs. Leith Mullings and Lisa Bowleg, who centered much of this research in the overlapping and interlocking oppression of structural racism and sexism (also known as gendered racism) (Laster Pirtle & Wright, 2021) on cisgender women racialized as Black. Given the overlapping and interlocking oppression of structural racism, sexism, and potentially homophobia, transphobia, and genderism in the lives of PWM that are racialized as Black and/or LatinX in Northern Manhattan, we thought it prudent that we apply intersectionality in this illustration. We used the concept of overlapping oppression to identify the groups with the greatest need for menstrual health equity intervention—PWM that are racialized as Black and/or LatinX. Further, we used intersectionality to identify key local areas where these groups live (e.g., Northern Manhattan), the structural determinants greatly impact our sample (i.e., healthcare access), and other determinants/circumstances at play (e.g., food access and literacy) that affect the menstrual health of PWM racialized as Black and/or LatinX. This led us to offer the intervention at housing associations. Thus, engaging intersectionality in the design process aided us in developing a structural intervention tailored to the needs of specific local communities.

#### Logic model

**Fig. 1:**
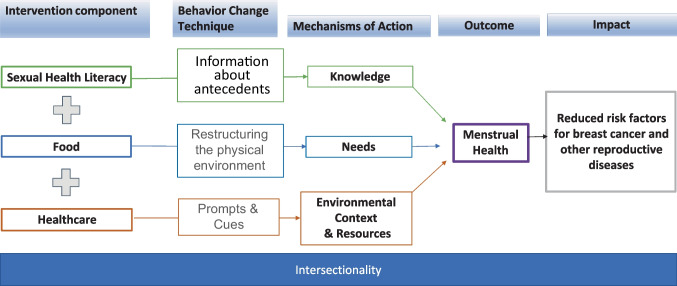
Logic model

We synthesized our positionality and theoretical framework to inform the logic model for this study. We also used the theory and technique tool developed by the Human Behavior Change Project (Michie et al., 2011) to align the intervention components with techniques and mechanisms of action substantiated in the literature and expert consensus. Studies that use techniques of *information about antecedents*, *restructuring the physical environment*, and *prompts and cues* show that mechanisms of action include *knowledge*, *needs*, and *environmental context and resources* (aspects of a person’s situation or environment that discourage or encourage a behavior) (Johnston et al., 2021). Our logic model (Fig. 1) illustrates three components of our intervention, which we hypothesize in combination make the intervention on menstrual health effective. Instead of typical research priorities examining additive and synergistic effects, we have prioritized providing resources equally across the community and have designed the study to test the delivery strategy of the healthcare system level component. While this approach descends from behavioral change literature, we maintained the emphasis on structural change and not individual-level behavior in response to critical race theory’s focus on structural causes of racism. As described below in the “Evaluating Mixed Methods in Clustered RCT” section, this logic model becomes the basis for our living logic model that will evolve over time as we incorporate more community views into the model. By incorporating the emic view into the logic model, we will be aware of shifts in our positionality and how the logic model may differ across intersecting social identities.

### Study Design

**Fig. 2:**
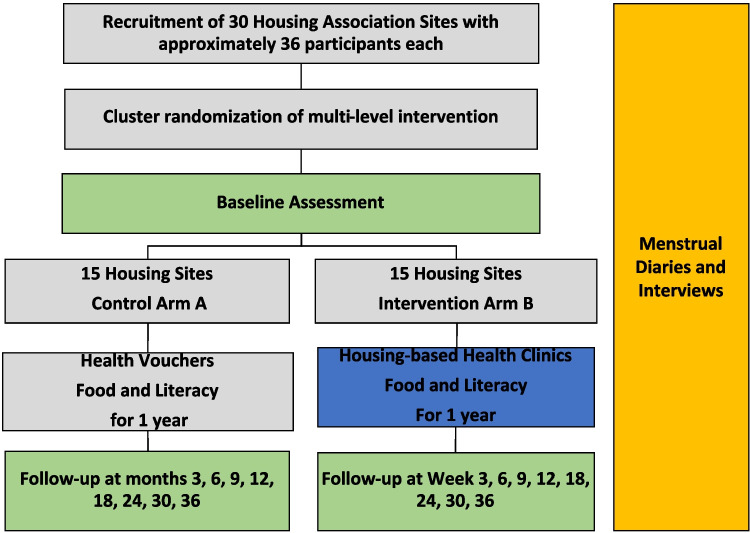
Embedded mixed-method, cluster RCT design

This illustrative example is a mixed-methods (Zhang & Creswell, 2013), parallel cRCT with 30 clusters randomized at the housing level. The intervention is a 1-year multilevel intervention that increases access to nutrient-rich food and sexual healthcare. Additionally, the intervention will increase literacy for a cross-section of participants on trauma and menstrual health and train healthcare providers on culturally responsive care. The study will be conducted at 30 New York City Housing Association (NYCHA) buildings in Washington Heights and Harlem. The combined demographics of all public housing developments in New York City is about 37% Black, 55% Hispanic, 3% White, and 3% Asian (NYU Furman Center, 2019). To effectively offer a structural intervention, community-based and multisector partnerships must be established in year 1, with the intervention occurring in staggered waves in years 2–4, and 1.5 succeeding years for in-depth follow-up and dissemination of the findings to the community and others at-large (see Fig. 2). We will conduct the study across five waves so that six buildings will be randomized at a time making recruitment and study logistics more feasible. We will stagger recruitment by 3 months between each wave.

We will use a mixed-method design that embeds qualitative methods within a quantitative cRCT. We will assess quantitative outcomes using surveys and menstrual tracking phone applications within the trial design. We will assess qualitative outcomes through diaries and in-depth interviews with community health workers and a subset of participants.

### Setting

We will randomize 15 buildings to Arm A, the control arm, which will receive free delivered groceries and psychoeducation at their individual housing associations and medical vouchers to attend pre-existing sexual health clinics outside of their buildings. Arm B, the intervention arm, will receive free delivered groceries, psychoeducation, and sexual health services at their building’s housing association. Housing associations inherently provide space and social network ties that can be leveraged for health promotion given multiple generations live together (Hernández, 2019). While housing-based health interventions overcome barriers such as transportation, travel time, and childcare, some individuals may be reluctant to participate in housing-based programs out of concerns for privacy (Hernández, 2019). Although social distance from neighbors (i.e., sticking to oneself and minding one’s business) can be an important safety strategy in precarious circumstances, strong social ties also confer safety benefits that also support health.

### Sample

The sample comprises of PWM from diverse family arrangements. We will include four groups of 240 PWM at different stages of the life course to expand the benefit of the intervention and to reduce the scientific burden of long-term longitudinal follow-up (Table 1). We will recruit pre-menarche youths, pre-parous PWM between 18 and 40 years who have not given live birth at baseline, postpartum PWM between 18 and 40 years who have given live birth that may be lactating, and perimenopausal PWM aged 40–55 at baseline. We will post flyers at the housing associations in Washington Heights and Harlem to recruit participants at each site. We will also work with the tenant association to recruit participants. We will recruit after randomizing buildings, but recruiters will be blinded to intervention status, and we will use the same recruitment and consent materials across arms (Hemming & Taljaard, 2023). When we randomize buildings, we will take into account geography so that buildings recruited during the same wave will not be physically near to each other.

**Table 1:** Methods to measure structural racism domains across the life course

**Population**	**Domain of structural racism**			**Method of data collection**	
	**Education**	**Food insecurity**	**Healthcare**	**Qualitative**	**Quantitative**
Premenarcheal girls	• Provide community-based culturally appropriate information on sexual health and aging • Use menstrual tracking apps as an educational tool	• Partner with food pantries to provide access to fresh, nutrient-rich foods • Provide community-based culturally appropriate information on cooking healthy foods	• Provide guidelines and training to pediatricians and obstetrics gynecologists on how to use data collected through menstrual tracking apps to help monitor menstruation as a vital sign • Provide vouchers or housing-based clinics	Weekly diary; quarterly interviews	Observation of age at menses and menstrual cycle characteristics
Pre-parous women with normal and abnormal menstrual cycles (years 18–40)				Weekly diary	Observation of menstrual cycle characteristics
Postpartum women				Weekly diary; quarterly interviews	Observation of menstrual cycle characteristics
Perimenopausal women				Weekly diary; quarterly interviews	Observation of age at menopause and menstrual cycle characteristics

### Multilevel Intervention Components

We will deliver interventions at two levels: literacy, healthy food access, and healthcare access at the community level (separately at each building), and healthcare training at the institutional level (Table 1). By providing access to psychoeducation, healthy food, and healthcare on different levels of the cRCT, we can test the effect of in-housing healthcare access compared to referral-based healthcare access, while controlling for other structural determinants (literacy and healthy food access) which we are keeping constant across arms.

#### Literacy

Researchers have called for anti-racist and anti-oppressive paradigms to guide the development and implementation of trauma-informed interventions (Adkins-Jackson et al., 2023; Griffith et al., 2007; Raskind, 2020). A trauma-informed intervention incorporates psychoeducation to help participants learn about the root causes of trauma and the breadth of its effects including hypervigilance and lack of trust (Phojanakong et al., 2020).

##### Procedure

We will hold monthly age-group specific and cross-age group psychoeducation sessions on trauma, sexual health, and menstrual health. PWM youth will meet the first week of the month, PWM adults the second week, PWM elders the third week, and everyone will meet the fourth week. We will provide community-based culturally appropriate information on the root causes of sexual and menstrual health as well as a comprehensive understanding of structural factors like racism and sexism. Community health workers will help to administer sessions and will work with buildings in both arms to avoid cross-classification (Cafri et al., 2015).

#### Food Access

Food deserts (limited access to nutrient-rich foods) and food swamps (abundant access to nutrient-deficit food) contribute to food insecurity for many previously redlined and segregated neighborhoods where a majority of racialized and minoritized groups reside (Mokiao & Hingorani, 2021; Morland & Evenson, 2009). Food insecurity not only plays a significant role in metabolic disorders like obesity and chronic kidney disease, but food insecurity can also influence menstrual health (Crews et al., 2014) as the hypothalamus-pituitary-ovarian axis is highly sensitive to fluctuations in energy availability as well as psychosocial stress (Ellison, 1994; Ellison et al., 1993; Nepomnaschy et al., 2007). New York City has many independently owned convenience stores that most racialized and minoritized communities rely upon (Hill, 2021). Though having such stores is associated with the prevalence of obesity (Morland & Evenson, 2009). While access largely drives food insecurity, affordability is a vital component with the combination of these factors being influential for New Yorkers (Herforth & Ahmed, 2015).

##### Procedure

We will partner with an established community food pantry to provide free access to fresh, nutrient-rich foods through grocery delivery to participants in all arms. We will also provide community-based culturally appropriate recipes. At a given time and day of the week, we provide free food to the community-at-large at each housing association building. Prepared bags of food will be set aside for participants in the study. Community health workers will help to distribute food.

#### Healthcare Access

Healthcare as a structural determinant of health refers to limited access to services like primary providers, specialists, clinical tests, and clinical trials (Baldwin et al., 2008; Gander et al., 2018; Paris Adkins-Jackson et al., 2021; Upadhyay et al., 2021). From as early as slavery in the US, access to healthcare for persons racialized as Native American, Black, and Asian have been scarce (Benjamin, 2021; Feagin & Bennefield, 2014). Limited access continued through Emancipation and required legal intervention to change through the Hill-Burton Act of 1946 and Indian Health Care Improvement Act of 1976, which expanded funding to hospitals to increase access for racialized and minoritized groups, though these institutions were and are too often underfunded (Bernard et al., 2017; Dowell, 1987). There were even laws that prohibited the expansion of businesses created by persons racialized as Asian (Lee, 2013). Thus, desegregation/integration provided racialized and minoritized groups the greatest access to healthcare services; however, the remnants of residential segregation linger (Schuyler & Wenzel, 2022).

##### Procedure

For the intervention arm at individual housing association buildings, we will offer monthly sexual health clinics through mobile units (many of which are currently serving Harlem). At the control housing associations, participants will be given vouchers to a permanent sexual health clinic. Participants that seek care either at the mobile sites or through the voucher will receive the culturally responsive care from a provider that has been trained for this study (see below). Community health workers will help to make clinic appointments.

#### Healthcare Training

The other aspect of healthcare as a structural determinant is the role of medical racism in the practice of healthcare (Matthew, 2015; McLemore et al., 2018). Medical practices are founded on scientific racism (Legha & Gordon-Achebe, 2022; Schmidt & Waikar, 2021; Washington, 2006) that undergirds medical training today (for the White Coats for Black Lives National Working Group et al., 2015; Green et al., 2022). Thus, the local healthcare system needs equal intervention. For PWM, gender biases in medicine compound medical and scientific racism producing greater barriers to access. Menstruation and menstrual-related disorders are under researched, medical education is lacking, and there is a history of clinicians dismissing PWM pains and menstrual complaints as normal (Ross et al., 2023).

##### Procedure

We will provide training to clinicians for all sexual health services (mobile included) that participants from the study may experience. Our culturally responsive clinician training will include a focus on how racism and sexism undergird processes that underdiagnose menstrual disorders. Clinicians will learn how to communicate health information in culturally responsive ways and support participants from diverse racialized groups with varying structural determinants of health. We will administer a pre-posttest survey to measure change in knowledge about gender- and race-based delivery of medicine.

### Variables

Table 2 summarizes our hypotheses, exposures, and outcomes for each intervention level.

**Table 2:** Data by hypothesis and intervention level

**Hypothesis**	**Intervention level**			
	**Individual**	**Community**	**Institutional**	**Structural**
**1**: Participant menstrual health via regular cycle length and later ages at menarche and menopause will be improved	*Exposure*: Literacy *Data*: Pre/post-assessment, diaries, interviews, use of tracking apps	*Exposure*: Literacy *Data*: Attendance at sessions		Multilevel intervention offered in housing association for PWM that experience intersectional oppression due to racism and sexism
	*Exposure*: Healthy food *Data*: Pre/post-assessment, diaries, interviews	*Exposure*: Healthy food *Data*: Attendance at weekly giveaways		
**2**: Participant access to sexual health services will increase	*Exposure*: Healthcare *Data*: Pre/post-assessment, diaries, interviews, use of healthcare location	*Exposure*: Healthcare *Data*: Autoethnographies of community health workers	*Exposure*: Healthcare *Data*: # providers trained in structurally and culturally responsive care; pre/post-assessment of participants	

#### Menstrual Healthcare Access Outcomes

Our primary process outcome will be the number of sexual health clinic visits in each cluster. Participants will provide their voucher number when booking and attending clinics. The sexual health clinics will provide a list of voucher numbers monthly over the course of the intervention. To assess structurally and culturally responsive care, we will use a pre/posttest design to survey participants about the healthcare they receive at baseline and follow-up period. The diaries and quarterly interviews may also document relevant information. We will engage the community health workers in autoethnographies to document their observations of healthcare during housing-based clinics as well as feedback from those using vouchers. In addition, we will interview participants about implementation outcomes such as fidelity during the quarterly interviews.

##### Menstrual Cycle

We will collect quantitative menstrual cycle data using a menstrual tracking smartphone application on a cellular device that we provide to all participants along with Internet access for the study duration. These data will include dates of menstrual bleeding that we will use to calculate menstrual cycle length, regularity, and menstrual blood flow. We will also assess age at menarche and menopause in those that were pre-menarche and perimenopausal at baseline through repeated surveys. Concurrently, we will ask PWM to keep menstrual diaries to share their experiences and perspectives with their cycles, menstrual health, and other experiences they are willing to share that contextualize study-related factor. Qualitative data will include structural and psychosocial stressors and their overall menstruation experience.

#### Other Outcomes

##### Literacy Outcomes

We will collect pre- and post-assessments of knowledge of sexual and menstrual health, as well as encourage participants to write in their diaries, participate in our quarterly interviews, and use menstrual tracking apps as a part of the curriculum. In addition, we will record the number of attendees per psychoeducation session.

##### Food Outcomes

We will collect pre- and post-assessments of food consumption and access using standard questionnaires to track. Additionally, we will encourage participants to write in their diaries and quarterly interviews, and we will record the number of times they attend our weekly food giveaways.

### Analysis

#### Quantitative Analysis

To assess the process outcome, we will compare the proportion of sexual health service visits between the two arms by performing a mixed-model logistic regression analysis, with building as a nested random effect. We will perform the same analysis for the attendance outcomes, and none of these outcomes has a pre-test measure. For the individual health outcomes, we will use linear mixed-effects models to study the intervention effects on menstrual cycle, literacy, and food outcomes, adjusting for pre-test values for each outcome. We expect to see equal improvement in the food and literacy outcomes, since both arms receive the same intervention on these two factors. Cluster-specific random intercept will be used to account for intra-class correlation. The model will be further adjusted for baseline covariates that are not balanced across treatment groups. We will perform frailty survival analyses for the age at menarche and age at menopause so that the hazard function appropriately accounts for the correlation within clusters.

##### Power

We performed the following power calculations using the clustersampsi command in Stata/SE 17.0 (Hemming & Marsh, 2013), assuming balanced and complete data. For our primary outcome, the proportion of sexual health service visits, we will be able to detect a difference of 0.14 in the intervention arm compared to the control arm if the proportion in the control arm is 0.3 with a total of 30 clusters of 32 people each and assuming 80% power, 0.05 significance, and intracluster correlation of 0.05. For menstrual cycle characteristics, we will compare the mean cycle length between the study arms. If the control arm had a mean cycle length of 34 days and a standard deviation of 4 days, we will be able to detect a 1.5-day change in menstrual cycle length with 30 clusters and 8 pre-parous or postpartum PWM, taking into account a intracluster correlation of 0.05. For menarche and menopause outcomes, we will use frailty survival models to estimate the effect of the intervention. We estimate a hazard ratio of 1.5 for a two-sample comparison of survivor functions in a cluster-randomized control trial with 30 clusters and 8 prepubertal or menopausal PWM in each cluster, with 80% power, significance at 0.05, and intracluster correlation of 0.05. While 30 clusters are on par with the average cluster size in cRCTs, we will correct for the small sample by using Residual (Restricted) Maximum Likelihood since type I error can be up ~ 7% for studies with 20–40 clusters. We will also adjust the test statistic with Kenward-Roger degrees of freedom if the cluster structures are balanced (Kenward & Roger, 2009). If the cluster sizes vary, we will use alternative small sample corrections.

#### Qualitative Analysis

After transcribing the diaries and quarterly interviews, we will use a grounded theory approach to derive major themes from the data, using Dedoose software where a team of four coders will use inductive and deductive coding. The analytic team will review the codes for major themes to examine the connections between themes.

##### Saturation

Unlike quantitative data, there is not a need to apply sample-specific findings to a population-level understanding of phenomenon, although there are concepts like saturation that mirror similar concepts in qualitative research. Saturation is the point at which no new themes are being observed in the data analyses (Saunders et al., 2018). Thus, participants drive the data available for qualitative analysis. Because qualitative analyses are often iterative and follow each interview, we can quickly determine whether there are missing perspectives that merit further expansion.

#### Triangulation of Quantitative and Qualitative Data

**Fig. 3:**
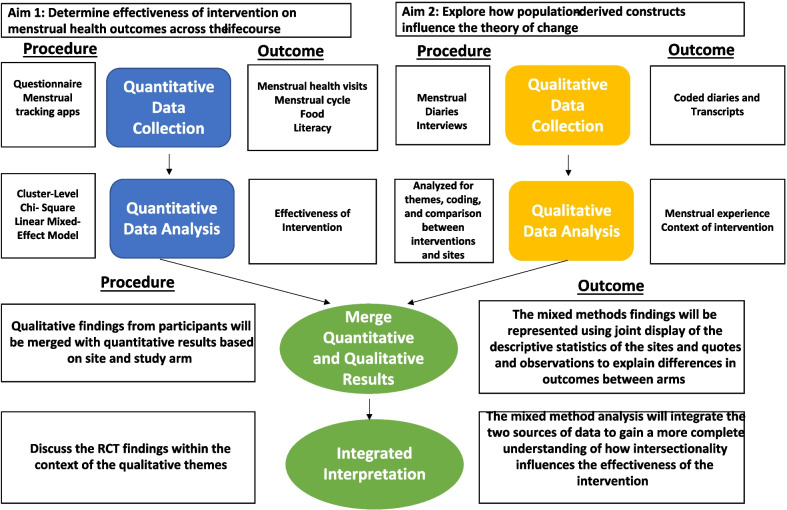
Integration within embedded mixed-method design

Figure 3 illustrates the integration of qualitative and quantitative data sources. All qualitative data (diaries, interviews) will be triangulated with quantitative data (frequency of session attendance, app use, food and healthcare service use based on participant, cluster, and study arm). We will use joint display to annotate descriptive statistics of the sites with quotes from interviews and diaries to explain differences in outcomes between arms.

##### Literacy and food

We will triangulate psychoeducation and food access attendance and pre/post-assessments with participant’s diaries and interviews by assigning codes related to knowledge, perspective, and practice across the qualitative data. Raters will examine whether there is consistency across qualitative and quantitative data on these codes.

##### Healthcare

For the healthcare access and menstrual health outcomes, we will have collected quantitative and qualitative data concurrently. Regarding the effectiveness of the trial to increase menstrual healthcare access, we will discuss the cRCT findings within the context of the qualitative themes we identify regarding intersectionality and implementation. The mixed-method analysis will integrate the two sources of data to gain a more complete understanding of how intersectionality influences the effectiveness of the intervention. We are intentionally not using quantitative methods to assess intersectionality, such as stratifying by racialized group, because intersectionality is not an additive or multiplicative experience.

## Deliverables

To determine the effectiveness of the multilevel cRCT on menstrual cycle outcomes, we must do so by level of structural impact: individual, community, and institutional. We will examine the individual-level change in literacy, food access, and healthcare utilization on sexual and menstrual health via maintenance of regular cycle length and later ages of menarche and menopause (for appropriate participants). Triangulation of pre/post-assessments with diaries and interviews and self-reported use of tracking apps will yield an understanding of what new psychoeducation knowledge has been retained and how it has been interpreted and implemented by participants. These data will also infer the degree to which menstrual health experiences were observed by participants after changes in food and healthcare access. This information can be used to infer impact of structural factors on menstrual health and health behavior.

Community-level change will be determined by examining triangulated data that includes number of attendees at psychoeducation classes and food giveaways to understand changes in literacy and healthy food access. These data will yield information about engagement with study components, which can be used to infer willingness, motivation, and commitment, in addition to the need for structural resources among the housing community-at-large. We will also examine change in healthcare access through the autoethnographic experiences of community health workers. This information will yield a robust perspective on the feasibility of the intervention and challenges to implementation and sustainability.

Institutional-level change will be examined using triangulated data from the number of providers trained in structurally and culturally responsive care and pre/post-assessments by participants about their healthcare experiences. These data will inform effectiveness of the provider training on their interpersonal interactions with participants. This information will aid in understanding the likelihood of returning for further healthcare services.

Through all these levels, we will understand the impact of literacy and healthy food and healthcare access on menstrual health. In addition, we will be able to determine if by addressing upstream factors, the benefits trickle down to participants. The diaries and interviews will be particularly useful in observing how each level interacts to produce structural impact. Further, the diaries and interviews and autoethnographies of community health workers will capture how structural impact specifically influences menstrual health for persons who experience intersectional oppression from racism and sexism. This level of specificity might not be captured in the pre/post-assessments, which focus on change in behavior and access over the study period. The diaries and interviews will offer longitudinal, contextualized information to inform the effectiveness of the intervention (Fig. 3).

### Evaluating Mixed Methods in Clustered RCT

We will use the qualitative data collected at baseline to identify additional variables to measure at follow-up visits. This will allow for the examining constructs that emerged from the population understudy that we as researchers did not hypothesize at study onset. To evaluate mixed methods in cRCTs, we will create a “living logic model.” Starting with the one we have proposed, we will revise our logic model diagram after quarterly interviews, either adding or removing variables, to show what is adapted after consulting with the community understudy. We will then compare our final statistical models with and without these community-derived variables to illustrate how including these variables impact the analysis of results. We will also contextualize the results of the intervention using the qualitative implementation results and will highlight what interpretations would not have been possible with quantitative methods alone.

## Strengths and Weaknesses

We have detailed a hypothetical study to illustrate how mixed methods can evaluate and enhance multilevel structural interventions to address health disparities. We now discuss the overall strengths and limitations through discussing four “balancing acts” researchers may encounter when applying our approach to other studies.

### Balancing Statistical Power with Feasibility

Clustering at the community level makes delivery of a multilevel intervention feasible, but the trade-off is reduced statistical power given the correlation of data within a cluster. However, our power calculations account for correlated data and indicate adequate sample sizes. In this hypothetical example, we assume balanced and complete data. Randomization, in theory, should reduce any confounding, but in practice, sample attrition could lead to residual confounding and should be factored into analyses.

### Balancing Rigor with Ethical Considerations

Typically, RCTs use contact or no-contact controls. Community-based cRCTs addressing disparities must consider the minimum resources offered to all participants based on equitable principles, and so no-contact controls are seldom ethically justified. Solutions to conducting ethically and rigorous studies include using a step-wedge design or to offer the intervention at smaller units of organization (i.e., individual and community levels) and only offer the system level intervention to one arm (healthcare service). An alternative is to offer interventions at all levels to both arms but alter the delivery of the intervention between arms. We chose the latter in this study because we felt it was equitable to offer all aspects of the intervention to everyone in the community. This may dilute the magnitude of effectiveness, but such dilution is also present in standard clinical RCTs that provide a placebo to the control arm when the effect of the treatment is the total effect minus the effect of the placebo. In addition to providing the intervention to all, it is also important to consider sustainability of what is provided. Partnering with existing community organizations with sustainable infrastructure to deliver intervention components after the study ends is important in the ethical conduct of community-level interventions.

Ethical considerations also come into play regarding efforts to reduce measurement error. Self-reported measures, such as those used to assess menarche, menopause, and cycle length in our illustrative example, are often assumed to introduce the potential for misclassification and measurement error. From some scientific perspective, a more objective and rigorous way to measure these outcomes would be to collect biospecimens for hormone measurement. Not only are these data subject to bias, but the burden and acceptability of these measures in diverse groups may not be feasible. Therefore, we suggest researchers perform validation studies on their more participant-friendly methods either before the main study or on a subset within the main study.

### Balancing Mechanistic Understanding with Effectiveness

In addition to the effectiveness of an intervention, there is a need to know how and why they work. The most rigorous design would theoretically test each component of a multilevel intervention in its own arm, but this is not an efficient design for structural determinants. Therefore, the traditional goal of testing one underlying mechanism must be weighed with the overall goal of equity. Since we hypothesize structural drivers matter more than individual ones, we use an efficient two-arm design to test only the mechanism of healthcare access delivery. This approach is efficient and equitable, though it does not distinguish underlying mechanisms of the food and literacy intervention components, though we can compare pre- and post-tests in both arms to estimate the effect of these components.

Cost-effectiveness is also important when considering the wider implementation of the intervention. It is likely that if the intervention is effective that there are potential healthcare costs savings either through prevention or early detection of disease. To be effective, these savings would need to be more than the costs; therefore, preliminary data on the study and intervention costs could be collected during this effectiveness phase.

### Triangulation

Using triangulation, such as measuring cycle length through recall, prospective diaries, and menstrual tracking apps, will also allow for researchers to examine measurement error and determine differential bias. Using mixed methods to triangulate different sources of data of the same construct is a robust methodology. This is due to the convergence of quantitative and qualitative data substantiating rigor. Divergence of results may indicate misalignment of quantitative and qualitative methods. Though the trade-off to this approach is that mixed methods may require more resources for a study than either method alone.

## Conclusion

Our multilevel intervention tackles structural determinants of menstrual health. Our study aligns with the NIMHD Minority Health and Health Disparities Research Framework, which recognizes interplay of factors across multiple levels including different domains and levels of influence (*National Minority Health and Health Disparities Research Framework*, n.d.). Our structural intervention addresses three domains of influence (behavioral, physical/built environment, sociocultural environment) and three levels of influence (individual, community, healthcare system) within the NIMHD framework. We have designed the intervention to serve all participants rather than a privileged subset, and we have embedded qualitative methods within a quantitative cRCT to capture what cannot be measured and expand on what can be. Such an approach relies on reflexivity of the researchers not only our positionality as we embark on the study but also our role as the intervention unfolds. We also see the community health workers as units of change and incorporate their perspectives as a major data point to inform interpretation of our findings. The living logic model will be the record of these processes that will serve to interpret the impact of the intervention and our proposed methodology and incorporate the perspective of the population understudy into the scientific investigation.
